# Design, Fabrication, and Testing of a Fully 3D-Printed Pressure Sensor Using a Hybrid Printing Approach

**DOI:** 10.3390/s22197531

**Published:** 2022-10-04

**Authors:** Akash Verma, Ruben Goos, Jurre De Weerdt, Patrick Pelgrims, Eleonora Ferraris

**Affiliations:** 1 Department of Mechanical Engineering, KU Leuven, Campus De Nayer, 2860 Sint-Katelijne Waver, Belgium; 2 EmSys Research Group, Thomas More Mechelen—Antwerpen, 2860 Sint-Katelijne Waver, Belgium

**Keywords:** biomedical pressure sensor, Aerosol Jet^®^ Printing, screen printing, selective laser sintering

## Abstract

Pressure sensing is not a new concept and can be applied by using different transduction mechanisms and manufacturing techniques, including printed electronics approaches. However, very limited efforts have been taken to realise pressure sensors fully using additive manufacturing techniques, especially for personalised guide prosthetics in biomedical applications. In this work, we present a novel, fully printed piezoresistive pressure sensor, which was realised by using Aerosol Jet^®^ Printing (AJP) and Screen Printing. AJ^®^P was specifically chosen to print silver interconnects on a selective laser sintered (SLS) polyamide board as a customised substrate, while piezoresistive electrodes were manually screen-printed on the top of the interconnects as the sensing layer. The sensor was electromechanically tested, and its response was registered upon the application of given signals, in terms of sensitivity, hysteresis, reproducibility, and time drift. When applying a ramping pressure, the sensor showed two different sensitive regions: (i) a highly sensitive region in the range of 0 to 0.12 MPa with an average sensitivity of 106 Ω/MPa and a low sensitive zone within 0.12 to 1.25 MPa with an average sensitivity of 7.6 Ω/MPa with some indeterminate overlapping regions. Hysteresis was negligible and an electrical resistance deviation of about 14% was observed in time drift experiments. Such performances will satisfy the demands of our application in the biomedical field as a smart prosthetics guide.

## 1. Introduction

In this modern world, we live in an ecosystem of sensors (of multiple types and for various purposes). Physical sensors (stress, strain, tactile, temperature, humidity, etc.), chemical sensors (liquid, gas, pH, etc.), and biological sensors (cell-based, biomolecule based, microbial, etc.) are a few examples [1,2]. 

There are several ways to manufacture sensors using lithographic processes along with special coatings, such as electroplating or lamination techniques, etc. [3,4,5,6,7]. Despite the small and compact sizes of the components, there exist some drawbacks, such as a large number of processing steps and the high cost of the equipment, which can be recuperated by mass production only [4,8]. Printed electronics (PE) is an emerging field with reduced prototyping costs and a shorter time to market, providing a form factor while maintaining high accuracy and performance. More specifically, PE is an umbrella of techniques that involve printing or deposition of a functional material over a substrate, offering the possibility of producing devices that are thin, flexible, lightweight, cost-efficient, and environmentally friendly [9,10]. This technological area involves multiple disciplines and expertise, from electronics to manufacturing engineering, material science, chemistry, physics, and biology. It has proven its ability in multiple industries with applications such as switches, sensors (pressure, strain, etc.) [11,12,13]; thin-film transistors (TFT) [14], antennas and RFID tags [15], energy harvesting and storage (organic solar cells and batteries) [16], displays (OLEDs) [17], and so on.

PE techniques are typically divided into direct and indirect methods. Indirect printing makes use of a mask or a screen to selectively deposit the functional ink on the target substrate. It suffers from a limited design versatility but can serve large batch production purposes. Roll-to-roll printing, flexographic printing, gravure printing, and screen printing are common examples, with screen printing among the easiest to implement.

Screen printing (SP) in particular is a technique of transferring ink onto a substrate (e.g., paper, glass, plastics, fabrics, etc.) by using a mesh screen. A squeegee (rubber blade) is moved across the screen, thereby filling the mesh openings with ink. The maximum printing resolution is tens of microns and the layer thickness of one pass ranges from a few μm to 100 μm. This printing technique uses high viscous (100–100,000 mPa.s) ink [13]. A broad range of functional materials can be deposited, such as metal inks (Ag, Cu, etc.), carbon-based inks, dielectrics, etc.

On the contrary, direct printing makes use of no mask and deposits the functional inks directly on the substrate through a nozzle according to a designed pattern. These techniques are also named mask-less methods. They provide extended designs and prototype flexibility [11]. Inkjet printing and Aerosol Jet^®^ Printing (AJ^®^P or simply AJP) are the most common ones; AJ^®^P has received increased attention in recent years due to its unique capabilities. 

Aerosol Jet^®^ Printing can print microscale features (down to 10 μm of in-plane resolution) with nanometric thicknesses (~100 nm to several mm), on (theoretically) any substrate (rigid, flexible, flat, curved, fibre-based, etc.), and with a large variety of functional inks, including metal and polymer nano-dispersion, biological fluids, and water-based solutions, whose viscosity can vary in the range of 1–1000 mPa.s. Typical applications are antennas, RFID, interconnects, 3D electrodes, LED, photovoltaics, and more recently, electrical and (bio-)chemical sensors with significant industrial and societal impacts [18,19,20,21,22,23,24,25]. AJ^®^P ranges from traditional printed electronic (PE) applications to advanced bioelectronic devices and 3D microscale printing. The use of AJ^®^P of collagen for tissue engineering applications is also a novel application [26,27].

In this work, we used SP and AJ^®^P to develop a fully printed pressure sensor applied on an additive manufactured and customised substrate to realise patient-specific biomedical solutions. The sensing principle is piezoresistive, owing to its simple read-out, easy implementation, and good performance [28]. The sensor was designed, manufactured, and characterised with respect to sensitivity, hysteresis, repeatability, and time drift. It can be applied as a force detector and/or guiding tool in prosthetic joints or personalised surgical guides. The substrate used for this sensor is produced by additive manufacturing (AM) via the selective laser sintering (SLS) technique. AJ^®^P was specifically chosen because of its ability to print on free-form substrates and design flexibility. The use of AJ^®^P was accompanied by screen printing to deposit the sensitive layers due to the lack of piezoresistive commercial solutions for AJ^®^P. Hence, the work also shed light on the limitations and capabilities of the Aerosol Jet^®^ technique next to the sensor findings. To the authors’ knowledge, there is no literature report on the use of Aerosol Jet^®^ Printing in the fabrication of a fully printed pressure sensor. This work reports the novel combination of a PE (AJP +SP) and AM (SLS) for a fully printed (AM + PE) pressure sensor, as such, combined printing techniques are termed “hybrid printing”. This paper also describes the fundamental background on printed pressure sensors that facilitate a deep educational value for other researchers of different fields and further support discussion and work insights. 

## 2. Background on Printed Pressure Sensors

According to Narakathu et al. [29], conventionally made silicon pressure sensors are often expensive, produced on a rigid substrate, and lack the properties required for various sensing applications. To overcome these problems, sensors can be fabricated using PE techniques that are thin, lightweight, flexible, and cost-efficient. 

In the field of printed electronics, the major pressure sensor types are piezoresistive-based, capacitive-based, piezoelectric-based, and triboelectric-based sensors. Each has a different working principle, as illustrated in [30,31]. In Table 1, a few previous works of printed pressure and force sensors are compiled in terms of key materials, printing method, the working principle, and measurement range and values. In this study, we investigated the characteristics of piezoresistive-based sensors in the detail due to their easy application, high spatial resolution, and simpler readout [30]. 

### 2.1. Piezoresistive Sensors

Piezoresistive sensors in particular rely on the ability of piezoresistive materials to provide a change in resistance when a force is applied. According to Valle-Lopera et al. [13], the resistance of the piezoresistive material is inversely proportional to the applied force. The electrical resistance will be in the range of mega ohms when no force is applied and decrease when the applied force increases. The piezoresistive layer is typically a conductive polymer, which is made by dispersing conductive nanoparticles into a non-conductive polymer matrix.

**Figure 1:**
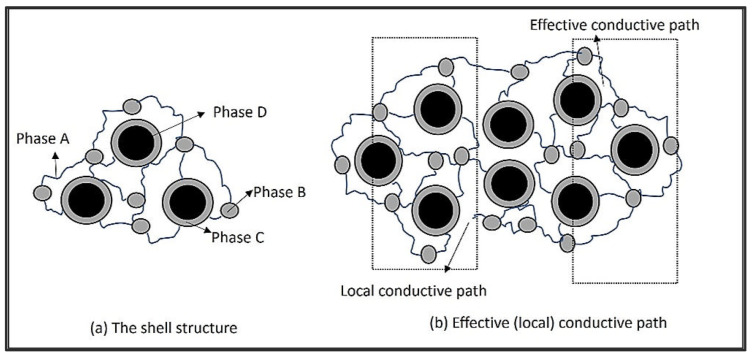
The schematic diagram for the shell structure where phase A is a rubber molecule chain, phase B is the crosslinking between the rubber chains, phase C is a macro-rubber that is absorbed by the carbon black surface, and phase D is the carbon black nanoparticle. (**a**) The effective (local) conductive path (**b**) of a nanocomposite [32].

Wang et al. [35] developed a pressure sensor where carbon black dispersed in a silicone rubber is used as a piezoresistive layer. Figure 1a shows the structure of this carbon black/silicone rubber nanocomposite, where phase A is a rubber molecule chain, phase B is the crosslinking between the rubber chains, phase C is a macro-rubber, which is absorbed by the carbon black surface, and phase D is the carbon black nanoparticle. Phases C and D act as framework, which is connected by elastic phases A and B which are form the background of the material [37]. When pressure is applied to the material, the gap between the carbon black particles decreases, resulting in the formation of local conductive paths. As shown in Figure 1b, an effective conductive path is formed where the local conductive path penetrates the outer insulating layer.

When the gap between carbon black particles is small enough to make them touch or come close to each other, two effects occur, i.e., the contact effect and the tunnelling effect, leading to the formation of a local conductive path. On the other hand, carbon black is mostly incompressible compared with non-insulating polymer matrix [37]. So, further compression can encourage translation and rotation of the carbon black particles, which can disturb these phenomena.

In summary, the following three steps in sequence can be seen [32]:

(1) Change in one effective conductive path: the applied pressure makes the gaps between two adjacent conductive particles smaller, which decreases the resistance of one effective conductive path. In particular, Kalantri et al. [38] estimated the tunnelling current (J) at an applied voltage (V) as follows:

(1) $$J=\frac{3\sqrt{2m\varphi }}{2s}{\left(\frac{e}{h}\right)}^{2}\exp\left(-\frac{4\pi s}{h}\sqrt{2m\varphi }\right)V$$

where m is the electron mass, e is the electron charge, h is the Plank’s constant, s is the particle distance within the insulating matrix, and φ is the height of the potential barrier between the adjacent particles.

The resistance R_m_ between two neighbouring filler particles can then be obtained as J/V, i.e.,

(2) $$R_{m}=\frac{J}{V}=\frac{2h^{2}s}{3e^{2}\sqrt{2m\varphi }}\exp\left(4\pi \sqrt{2m\varphi }\frac{s}{h}\right)$$

As shown in Equation (2), the decrease of *s*, caused by the pressure applied, leads to a decrease of R_m_ due to the decreasing distance between two conductive particles; hence, the tunnelling current increases while the resistance of one single effective conductive path decreases.

(2) Formation of effective conductive paths: the further compression makes the gaps between carbon black particles even smaller, leading to contact of filler particles and the formation of multiple effective conductive paths.

(3) Destruction of effective conductive paths: the further compression induces transverse slippage of the carbon black particles, which leads to the destruction of effective conductive paths, eventually compensated by the formation of other effective conductive paths.

$R_{m}\;$ can be further combined with the resistance across a conductive filler particle, R_c_ (intrinsic particle resistance), and generalised for a number of conductive paths of multiple particles in the total conductive polymer resistance R_t_, as follows [38,39]:

(3) $$R_{t}=\frac{\left(L-1\right)R_{m}+{\mathrm{LR}}_{c}}{S}\approx \frac{L\left(R_{m}+R_{c}\right)}{S}$$

where L is the number of particles forming the conductive path, and S is the number of effective conductive paths.

By further assuming (L−1) ~L and R_c_ to be negligible (as the resistance of the conductive filler particles is very low) [37], the resultant R_t_ (upon compression) divided by the initial resistance R_0_ (without compression and for an initial default polymer matrix particle distance equals to s_o_) results in the relative resistance formulated as:

(4) $$\frac{R_{t}}{R_{0}}=\frac{s}{s_{0}}\exp\left[-\gamma \left(s_{0}-s\right)\right]$$

where the constant $\frac{4\pi \sqrt{2m\varphi }}{h}$ is here indicated by γ and s indicates the generic polymer matrix particle distance upon compression and smaller of s_0_. By substituting the values of s_0_, s, and γ as defined in [38], Equation (4) results in:

(5) $$\frac{R}{R_{0}}=\left(1-\frac{\sigma }{E}\right)\exp\left\{-\gamma D\left[{\left(\frac{\pi }{6\vartheta }\right)}^{\frac{1}{3}}-1\right]\frac{\sigma }{E}\right\}$$

Hence, the relative resistance can also be expressed as a function of the applied stress (σ), the elasticity modulus of the polymer matrix (E), the conductive filler diameter (D), the filler volume fraction (ϑ), and the constant for the energy barrier of the polymer (φ).

(6) $$\frac{R}{R_{0}}=f\left(\sigma ,E,D,\vartheta ,\varphi \right)$$

## 3. Materials and Methods

### 3.1. Inks and Substrates

The substrate is a 3D (selective laser sintering—SLS)-printed polyamide (PA) board with dimensions of 7 × 7 cm, a thickness of 1 cm, and an average roughness Ra of 6 μm and Rz of 31 µm. The melting temperature is between 172 and 180 °C. This substrate was produced by Materialise, Leuven, Belgium. Grinding and polishing of the substrate were done using 600 and 2400 grit paper to smoothen the top surface to prevent defects during printing of the functional inks, while improving the adhesion and accurate deposition with limited spreading.

Two types of inks were used to produce the sensors. The first type was conductive silver ink (Metalon^®^ JS A221E, Novacentrix, Inc.), which was used to print the conductive paths. The second type was a piezoresistive ink (Carbon EMS CI 2050, ECM), which was used to print the piezoresistive pressure elements. The properties of the inks are given in Table 2.

### 3.2. Sensor Design and Fabrication

**Figure 2:**
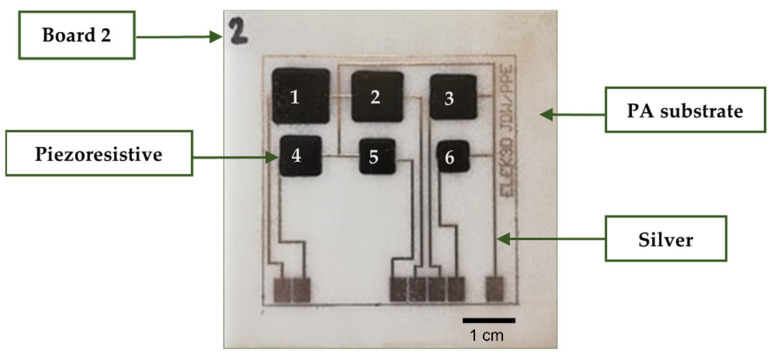
Arrangement of six piezoresistive pressure sensors printed on a PA substrate, with a sensing area decreasing from 100 to 25 mm^2^. The sensors are enumerated and ranked, ranging from 1 to 6, where “sensor PA_1” has the largest sensing area and is located in the upper left corner of the PA substrate.

Regarding the top of the PA substrates, a configuration of six sensors was produced with dimensions ranging from 100 to 25 mm^2^ and a typical thickness in the range of 30 μm, to allow for exploring the most reliable sensor size (Figure 2). A limited sensor dimension is here important to investigate, taking into account that multiple sensing elements (from 3 to 6 in count) will be applied on the prosthetic and/or guide surface to ensure optimal positional accuracy. Three samples of the sensor configuration were made to check for repeatability and to mitigate uncertainty. The sensor circuit consists of Aerosol Jet^®^-printed silver lines and pads, which act as interconnects, whereupon piezoresistive electrodes are screen-printed to provide the active material, from which the resistance will change in the function of the applied pressure.

In detail, the production process of the sensor board included the following steps. First, the conductive lines were Aerosol Jet^®^-printed onto the substrate, using Metalon^®^ JS A221E ink and the Optomec 300 series Aerosol Jet^®^ printer. Atomization of the ink (~1 mL) was reached by using the ultrasonic method. Nitrogen gas was used as inert gas to carry and collimate the aerosol, and a 300 μm diameter ceramic nozzle was adopted. Table 3 lists the print parameters. Next, thermal sintering of the printed pattern was conducted in an oven at 150 °C for 2 h.

**Figure 3:**
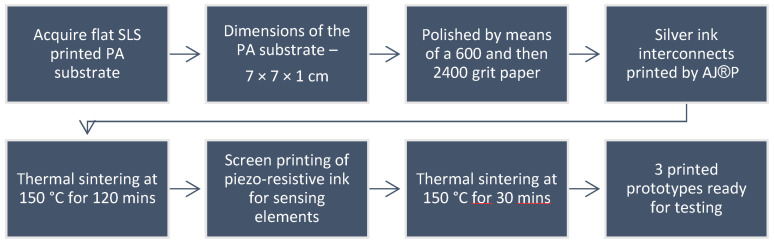
Process flow for the sensor’s prototype manufacturing.

Secondly, the piezoresistive pressure elements were manually screen-printed (by using a stainless-steel stencil), using the Carbon EMS CI 2050 ink with the help of a squeegee. According to the data from the ink supplier and the experiments conducted on the PA substrate, an optimal ratio of 60 wt% CI-2050 HR: 40 wt% CI-2050LR was found. This viscous ink was hand-stirred for a minimum of 5 min. Two layers were printed with a 30 μm thick stainless-steel screen to reach uniformity and avoid unwanted gaps in the printed pattern. The screen and the squeegee were cleaned with MEK (ketone) solvent. Next, thermal sintering was applied in an oven at 150 °C for 30 min. Figure 3 provides the process and production flow for the sensor’s prototype manufacturing.

All print steps were performed at room temperature and relative humidity of ~ 50%.

The Weiss Technik Heraeus thermal oven was used to sinter the printed lines. By adjusting the temperature of the oven or altering the sintering time, different sintering conditions can be achieved. The quality of the printed lines was inspected by employing a Hirox KH 8700 digital microscope so that critical information about the quality of the printed patterns before and after sintering could be obtained (overspray, presence of cracks, line width for sensor design, etc.).

### 3.3. Sensor Testing

The sensors were tested with respect to sensitivity, hysteresis, reproducibility, and time drift.

The loading signals were applied using an Instron 3367, a mechanical testing system that can perform high-quality tensile, compression, and bending tests. An Instron 2530-5 kN load cell (accuracy of ± 10 N) was then mounted onto the crossbeam of the Instron 3367 to monitor the applied load and allow for calibration of the zero-offset before each measurement. Before testing, the complete setup was also calibrated. The applied load was registered with a sampling rate of 50 Hz. The load was then transferred using a compression head, mounted onto the load cell, and equipped with a 1 mm thick rubber patch, to compensate for alignment errors and allow the application of a constant pressure across the whole sensitive area of each tested sensor. Vulcanised rubber was used as a patch material because of its low hysteresis and high elastic properties. Test conditions were conducted in force controlled. Specifically, in order to determine the change in resistance for a given signal and identify the sensor sensitivity, a ramping force was first applied from 0 to 125 N and then back to 0 N, with a force rate of 3 N/s. This signal corresponds to a pressure range of 0–1.25 Mpa and a rate of 30 Kpa/s. Comparing the change in resistance for an increasing and decreasing force also gives information about the hysteresis of the sensors. Next, each sensor was exposed to a cycling load, of the type 0–125–0 N, 50 cycles, with a force rate of 10 N/s, (i.e., a pressure range of 0–1.25 Mpa-0 and a rate of 100 Kpa/s) to determine the degradation of the sensing properties with increased use of the sensor. Finally, a constant load was applied at 125 N (1.25 Mpa) over a given period of time (10 min), to study the stability of the sensor in the function of time, also known as time drift. For each test, three measurements (repetitions) were conducted on each sensor of the three boards, except for the cyclic and constant load experiments, which were conducted on sensor one (S1) only. Between each repetition, a sensor was given 30 min to reach its initial condition. In all three cases, the load rates were small enough to consider the system in the quasi-static range [40,41]. Table 4 summarises the sensor testing methodology.

Finally, a Rigol DM3068 digital multimeter was adopted to measure the change in resistance to the applied force. Because of the difference in the sampling rate between the Instron 3367 (50 Hz) and the Rigol digital multimeter (2 Hz), the number of the recorded force data was 25 times higher than that of the change in resistance over the same period but they were merged and are displayed together.

**Figure 4:**
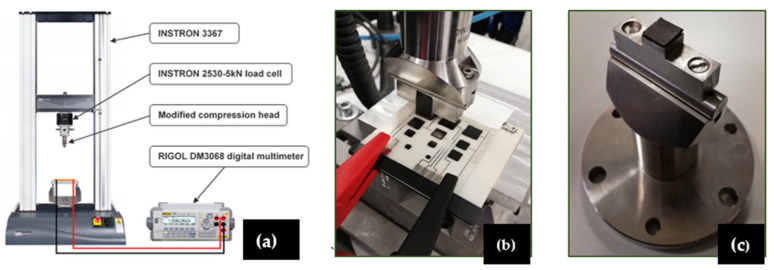
(**a**) Picture of the experimental bench connected with a digital multimeter for sensor testing; (**b**) non-conductive tape and crocodile clamps; (**c**) rubber patch on the compression head.

Figure 4 shows pictures of the experimental setup.

## 4. Results

### 4.1. Ramping Load, Sensor Sensitivity, and Hysteresis

**Figure 5:**
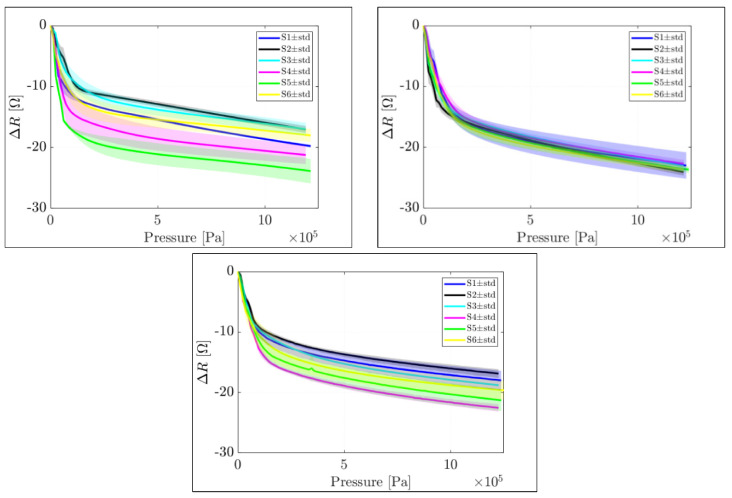
Change in resistance (*ΔR*[Ω]) vs. an increasing load per sensor dimension and replicas. Three figures signify three different manufactured sample boards.

Figure 5 plots the measured change in resistance (*ΔR*[Ω]) versus an increasing ramping pressure per sensor dimension and sample replicas over the three boards. A zero-set was applied to each sensor signal to compensate for the parasitic resistance, as a function of the initial sensor conditions (R_0_) and intrinsic particle resistance (R_c_). The pressure signal was calculated as the increasing force ramping signal (from 0 to 125 N, see Section 3.3) divided by the area of the sensing layer. Each plot in Figure 5 refers to each manufactured board. Each board contained six curves that corresponded to the six sensing elements. Each curve was a mean of three repetitions. The shaded region shows the standard deviation of the response of each sensing element on each board.

The sensor response in absolute change in resistance (*ΔR*[Ω]) varied as a function of the applied pressure range; specifically, two areas could be distinguished: a highly sensitive linear region at low-pressure data (range within 0.12 Mpa) with the grand mean sensitivity of 106.79 ± 15.46 [Ω/Mpa] and a low sensitive region for a higher load (range from 0.12 Mpa to 1.25 Mpa) with the grand mean sensitivity of 7.65 ± 0.81 [Ω/Mpa]. Table 5 also summarises the average sensitivities calculated per each sensor size in the high and low sensitive regions, for all the boards along with their standard deviation. Board 2 shows the most stable behaviour with the least standard deviation.

The statistical approach, of one-way ANOVA (analysis of variances), was used to determine whether the dimension or the location (different boards) of the samples had a statistical significance on the results. The factor dimension was analysed via six levels (six different dimensions of the sensing layer), while the board factor was analysed via three levels (from the boards manufactured). No direct relation was found between the dimensions of the sensing layer and the sensitivity response as a *p* value of *p=* 0.202 (*p >* 0.05) was determined, and similarly for the choice of the board, being *p=* 0.740 (*p >* 0.05) with a confidence interval (C.I.) = 95%; α = 0.05. Due to the reported mentioned *p* value, we failed to reject the null hypothesis (h_0_ = u_1_ = u_2_ = … = u_k_).

**Figure 6:**
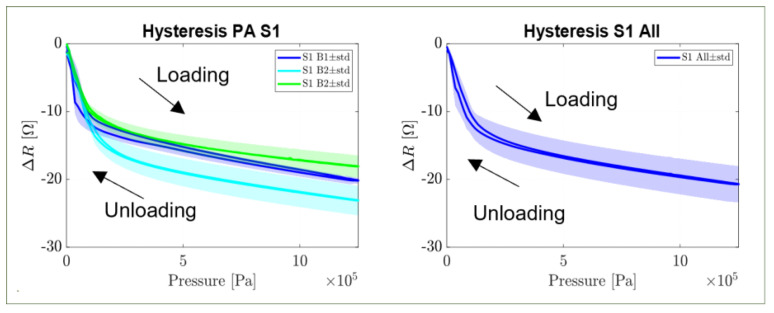
Hysteresis of sensor 1 (board sample (B1, B2, B3) (**left**) and average behaviour of all boards (**right**)) as the area comprehended in the resistance curve in the functions of loading and unloading signals.

Figure 6 shows the hysteresis data measured by applying a loading and unloading signal (ramping test of the type 0–125–0 N; i.e., 0–1.25–0 Mpa). The data are reported for the largest sensors (sensor 1) only and the average behaviour, as representative of the general response. The sensor hysteresis was minimal in one cycle and only presented in the high sensitivity region.

### 4.2. Time Drift Testing

**Figure 7:**
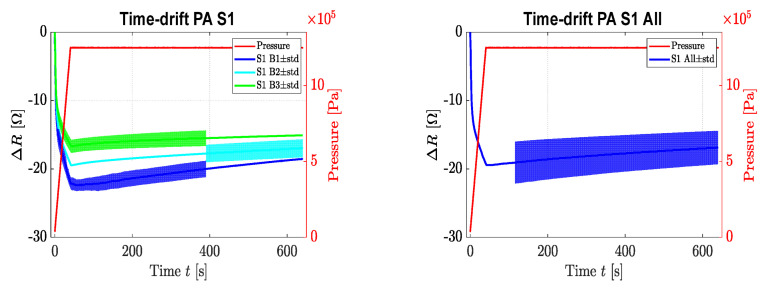
Time drift vs. change in resistance at 125 N in the function of time and maximum hold time of 10 min (sensor 1 (board sample (B1, B2, B3) (**left**) and average behaviour of all boards (**right**)).

During the time drift test, sensor one of each board was exposed to a force that increased from 0 to 125 N (0–1.25 MPa) with a compression rate of 3 N/s (30 KPa/s). Afterward, the force was kept constant at 125 N over a period of 600 s. The change of resistance in the function of time is illustrated in Figure 7, along with the combined time drift data of sensor one for all boards combined. Around 13% of the change of resistance (*ΔR* [Ω] vs. s) for 600 s was reported. No longer time duration was needed due to the demand of our application.

### 4.3. Cyclic Forces

**Figure 8:**
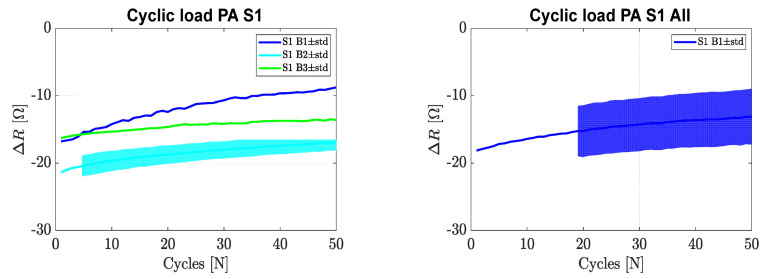
Cyclic force vs. change in resistance in the function of loading cycles (sensor 1 (board sample (B1, B2, B3) (**left**) and average behaviour of all boards (**right**)).

During the cyclic force test, sensor one of each board was exposed to 50 loading and unloading cycles where the load increased and decreased between 0 and 125 N (0–1.25 Mpa) at a compression rate of 10 N/s (100 Kpa/s). The change of resistance in the function of the number of cycles is illustrated in Figure 8. In this case, a change of resistance with respect to time (*ΔR* [Ω] vs. cycles) of 28.3% over the 50 cycles was observed.

## 5. Discussion

A new, customised, and fully printed pressure sensor for biomedical applications was designed and fabricated by Aerosol Jet^®^ Printing and screen printing, along with its testing performance in the different domains.

As from the results, the sensor responses highlighted two different sensitivity regions: (i) a highly sensitivity region in the range 0–0.12 MPa, characterised by an averaged sensitivity of 106.79 ± 15.46 Ω/MPa; and (ii) a low sensitivity region, within 0.12–1.25 MPa, characterised by an averaged sensitivity of 7.65 ± 0.81 Ω/MPa, i.e., about 14 times smaller. A procedure of zero-offset was applied to each sensor to compensate for the variation in parasitic resistance. The two regions also overlapped each other into a non-linear transition zone, here determined to take place at around 0.12 MPa. Accordingly, the same sensor design can be fine-tuned by the user for different demands and working conditions. As from the results of Table 5, the low sensitivity region, on the other hand, provides a more stable behaviour, with result variations up to ~10% against 15% of the high sensitivity region. Additionally, from the ANOVA data, the effect of the sensor size and manufacturing variability were found not statistically significant. This gives freedom to the user to select the most appropriate sensor dimensions, depending on the given constraints on size and required accuracy.

For the sake of the presence of two sensitivity regions with a transition zone, the sequences of the phenomena described in Section 2 can be called into question [42]. As from Equations (2) and (4), the (relative) material resistance exponentially decreases with the decrease of the particle distance until its contact upon compression (high sensitivity region). Due to the incompressibility of the conductive particles, further compression induces slippage and/or bouncing of the fillers within the elastic matrix, which disturbs the effectiveness of the conductive paths generated, and the system slowly approaches saturation (low sensitivity region).

The observed variations in the sensor responses and initial conditions can instead be ascribed to the differences in material properties across various batches. As from Equation (6), the resistance of the piezoresistive material can indeed also be described as a function of the diameter of the conductive fillers as well as their volume fraction (concentration) in the polymer matrix, whose variability can be significant from batch to batch. In this context, the inks were prepared manually due to the high viscosities of the components to be mixed; the screen-printing process was also applied manually. Lastly, the lengths of the interconnects of a sensing layer to the bond pads (where electrodes were placed for resistance recording) were different for each sensor [35,42].

Further tests showed that the change in resistance was higher during the loading cycle than during the unloading cycle in one performed cycle. This phenomenon is called the hysteresis of the sensor and can be seen as the area inscribed by the loading and unloading curves. As expected, this phenomenon is more pronounced in the region of high sensitivity, although only in small amounts, due to the higher impacts that small deformations of the reference conditions have on the particle distributions and their relative distances. On the other hand, such small deviations did not impact the proposed applications, and they could be ignored for the purpose of a guide in prosthetics for biomedical applications.

Time drift results revealed that the change in resistance decreased 13.16 ± 3.84% over a period of 600 s. This was a rather high drift as compared to the literature where time drift values of 5% were reported. During the time drift, relaxation of the composite occurred, resulting in better stability of the polymer matrix. This resulted in improved stability, as can be seen in Figure 7, where the change in resistance seemed to have a near-constant value. The reasons for such a drift could be structural tension of the top surface after the application of force on the sensing layer that the drift caused in the clamps during the readout, or the non-uniform stress application, in which there was an attempt to mitigate using an elastic rubber stamp on the head of the compression head [28]. Moreover, as mentioned in Section 2.1, the conduction path made by filler particles in the polymer matrix started to break with a longer duration of compression, or a further increase in the compression could cause this deviation in these measurements [35,38].

Figure 8 also shows the combined cyclic data of sensor one for all boards combined. When looking at the results, we can see that the change in resistance decreased 28.34 ± 16.95% over a period of 50 cycles. This is a rather high change in resistance as compared with the literature where a change in resistance of 8% was reported under cyclic loading. During the cyclic loading, relaxation of the composite occurs, resulting in better stability of the polymer matrix. This so-called ‘mechanical training’ results in improved stability and repeatability of the sensor. Such improvement is visible in Figure 8 as the curve approached a constant value. For our application, the demand for such vigorous dynamic cyclic testing was not necessary and, therefore, the above results can be well accepted.

## 6. Conclusions

Fully printed piezoresistive pressure sensors were developed on a SLS-printed substrate flat PA (polyamide) substrate using Aerosol Jet^®^ Printing (AJ^®^P) along with manual screen printing. Silver interconnection using silver nanoparticle ink (AgNPs) was printed by AJ^®^P while sensing piezo-resistive ink (carbon black) was manually screen-printed. The backgrounds of piezo-sensitive sensors have been well reported, and the working mechanisms were also described. To obtain a better understanding of the characteristics of the sensor, uniaxial compression testing was conducted on the Instron 3367 mechanical testing system, where the change in resistance (Ω) was measured in function of the applied pressure (in MPa). With the application of pressure, the absolute resistance (Ω) decreased (as expected and was recorded). Experiments were conducted to investigate the sensitivity, hysteresis, time drift, and cyclic testing response of the sensor. Sensitivity (*ΔR* [Ω]/MPa), in this work, was explained as the change of the electrical resistance (Ω) of a sensor in the function of applied pressure (in MPa). From the results of these experiments, we can conclude that the piezoresistive sensors have modes of high sensitivity ranging from 0 to 0.12 MPa with a sensitivity of 106.7 (*ΔR* [Ω]/MPa), where there also is a slight amount of hysteresis, as opposed to a second mode with low sensitivity, which ranged from 0.12 to 1.25 Mpa, with a sensitivity of 15.46 (*ΔR* [Ω]/MPa), which sufficed its demand for biomedical applications. There was a region of overlap between the two regions where the sensitivity gradually changed from a high to a low region. With the ANOVA testing, it can be concluded that the sensor size does not influence the sensitivity of the sensor. This was considered an advantage as we can use this methodology to print any desired applications with variable sizes. It is possible by changing the screen parameters of the screen printer. A similar printed sensor on a free-form SLS-printed substrate for the biomedical application has also been prototyped in the other work. Furthermore, to the author’s knowledge, it is a novel, fully printed (i.e., combining AM and PE techniques) pressure sensor. 

Such sensors can also be used in the future for posture recognition, with the matrix of sensor arrays. Moreover, they can extend their applications for in-sole pressure monitoring in shoes for gait analysis, or in mattresses and wheelchairs for patients experiencing reduced mobility. Furthermore, they can be implemented on curved or free-form surfaces. For future perspectives, sensitivity can be improved along with more extended measuring and sensing ranges.

## Figures and Tables

**Table 1:** Overview of printed pressure and force sensors along with materials used, printing techniques, and working range.

Working Principle	Materials Used	Production Method	Working Range	Ref.
Capacitive	Conductive: Silver Sensing: Polymer ink Top Layer: UV cured insulator Substrate: PBT	Inkjet Aerosol Jet		Polzinger [32]
Piezoelectric	Conductive: Silver Sensing: PVDF-TrFE Top layer: Silver Substrate: PET	Screen	Normal mode: 2pC/N Bend mode: 200 nC/N	Rajala [33]
Piezoresistive	Conductive: Silver Sensing: PDMS + MWCNTs Substrate: Kapton	Inkjet	Range: 2.5 to 640 kPa	Ramalingame [34]
Piezoresistive	Conductive: Silver Sensing: Carbon black Substrate: PET	Screen	Range: 1 to 100 N	Ahmad [28]
Piezoresistive	Conductive: Sensing: Carbon+ silicon		Range: 0–1 MPa	Wang [35]
Capacitive	PDMS, SWCNT, Air Gap	Spray coating. Mould	Sensitivity: 0.7 KPa^−1^ (for *p* < 1 kPa)	Park [36]

**Table 2:** Properties and details of the ink used.

Name	Metalon^®^ JS A221E	Carbon EMS CI 2050
Deposition technique	Ultrasonic AJ®P	Screen printing
Type	Conductive silver ink	Piezoresistive carbon ink
Solid content	40–60 wt% Ag	31 wt% Carbon
Other content	3–10 wt% 2,2-oxybisethanol 2–10 wt% isopropyl alcohol	Trade Secret
Dynamic viscosity	10–20 mPas	2500 mPas
Average particle size	35 nm	<5 µm
Cure temperature and time	150 °C for 120 min	150 °C for 30 min

**Table 3:** Printing parameters for the Optomec 300 series.

Parameter	Value
Atomizer gas flow	65 sccm
Sheath gas flow	45 sccm
Print speed	7 mm/s
Table temperature	23 °C
Number of layers	7

**Table 4:** Details of the sensor testing methodology.

	Ramping Force	Cyclic Force	Constant Force
Tested sensor (S)	S1–S6	S1	S1
Min force (N)	0	0	0
Max force (N)	125	125	125
Pressure range (Mpa)	1.25	1.25	1.25
Load rate (N/s)	3	10	-
Pressure rate (Kpa/s)	30	100	-
# cycles (/)	/	50	/
Holding time (min)	/	/	10
Repetitions	3	3	3

**Table 5:** Mean sensitivity and standard deviation for the high sensitivity region (HSR) and low sensitivity region (LSR).

#	Board 1		Board 2		Board 3		Mean +/− STD (Ω/MPa)	
	HSR	LSR	HSR	LSR	HSR	LSR	HSR	LSR
**1**	94.99	7.04	95.44	7.67	83.50	6.46	**91.31 ± 5.52**	**7.05 ± 0.49**
**2**	83.96	7.21	119.49	8.45	86.29	6.62	**96.58 ± 16.22**	**7.42 ± 0.76**
**3**	102.22	8.54	104.68	8.18	117.82	9.07	**108.25 ± 6.84**	**8.59 ± 0.36**
**4**	134.68	6.17	94.70	8.46	123.27	8.35	**117.55 ± 16.81**	**7.66 ± 1.05**
**5**	126.24	6.26	105.12	8.24	116.64	8.93	**116.00 ± 8.63**	**7.81 ± 1.13**
**6**	118.82	6.46	98.12	7.39	116.41	8.13	**111.11 ± 9.24**	**7.32 ± 0.68**
**Mean +/− STD (Ω/MPa)**	**110.15 ± 19.56**	**6.95 ± 0.88**	**102.92 ± 9.26**	**8.07 ± 0.43**	**107.32 ± 17.57**	**7.93 ± 1.12**	**106.79 *±* 15.46**	**7.65 *±* 0.81**

## References

[1] Cheng M., Zhu G., Zhang F., Tang W., Jianping S., Yang J., Zhu L. (2020). An review of flexible force sensors for human health monitoring. J. Adv. Res..

[2] Ni Y., Ji R., Long K., Bu T., Chen K., Zhuang S. (2017). A review of 3D-printed sensors. Appl. Spectrosc. Rev..

[3] Frutiger A., Muth J.T., Vogt D.M., Mengüç Y., Campo A., Valentine A.D., Walsh C.J., Lewis J.A. (2015). Capacitive soft strain sensors via multicore–shell fiber printing. Adv. Mater..

[4] Tseng P., Murray C., Kim D., Di Carlo D. (2014). Research highlights: Printing the future of microfabrication. Lab Chip.

[5] Kasani S., Curtin K., Wu N. (2019). A review of 2D and 3D plasmonic nanostructure array patterns: Fabrication, light management and sensing applications. Nanophotonics.

[6] Da Vià C. (2014). 3D sensors and micro-fabricated detector systems. Nucl. Instrum. Methods Phys. Res. Sect. A Accel. Spectrometers Detect. Assoc. Equip..

[7] Puers R. (1993). Capacitive sensors: When and how to use them. Sens. Actuators A Phys..

[8] O’Neill P.F., Ben Azouz A., Vázquez M., Liu J., Marczak S., Slouka Z., Chang H.C., Diamond D., Brabazon D. (2014). Advances in three-dimensional rapid prototyping of microfluidic devices for biological applications. Biomicrofluidics.

[9] Khan Y., Thielens A., Muin S., Ting J., Baumbauer C., Arias A.C. (2020). A New Frontier of Printed Electronics: Flexible Hybrid Electronics. Adv. Mater..

[10] Chang J.S., Facchetti A.F., Reuss R. (2017). A Circuits and Systems Perspective of Organic/Printed Electronics: Review, Challenges, and Contemporary and Emerging Design Approaches. IEEE J. Emerg. Sel. Top. Circuits Syst..

[11] Cruz S.M.F., Rocha L.A., Viana J.C. (2018). Printing Technologies on Flexible Substrates for Printed Electronics. Flexible Electronics.

[12] Woo S.J., Kong J.H., Kim D.G., Kim J.M. (2014). A thin all-elastomeric capacitive pressure sensor array based on micro-contact printed elastic conductors. J. Mater. Chem. C.

[13] Valle-Lopera D.A., Castaño-Franco A.F., Gallego-Londoño J., Hernández-Valdivieso A.M. (2017). Test and fabrication of piezoresistive sensors for contact pressure measurement. Rev. Fac. Ing..

[14] Lu S., Cardenas J.A., Worsley R., Williams N.X., Andrews J.B., Casiraghi C., Franklin A.D. (2019). Flexible, Print-in-Place 1D-2D Thin-Film Transistors Using Aerosol Jet Printing. ACS Nano.

[15] Machiels J., Verma A., Appeltans R., Buntinx M., Ferraris E., Deferme W. (2021). Printed Electronics (PE) As An enabling Technology To Realize Flexible Mass Customized Smart Applications. Procedia CIRP.

[16] Kopola P., Zimmermann B., Filipovic A., Schleiermacher H.F., Greulich J., Rousu S., Hast J., Myllylä R., Würfel U. (2012). Aerosol jet printed grid for ITO-free inverted organic solar cells. Sol. Energy Mater. Sol. Cells.

[17] Tait J.G., Witkowska E., Hirade M., Ke T.H., Malinowski P.E., Steudel S., Adachi C., Heremans P. (2015). Uniform Aerosol Jet printed polymer lines with 30 μm width for 140 ppi resolution RGB organic light emitting diodes. Org. Electron..

[18] Wilkinson N.J., Smith M.A.A., Kay R.W., Harris R.A. (2019). A review of aerosol jet printing—A non-traditional hybrid process for micro-manufacturing. Int. J. Adv. Manuf. Technol..

[19] Secor E.B. (2018). Principles of aerosol jet printing. Flex. Print. Electron..

[20] Seiti M., Ginestra P.S., Ferraro R.M., Giliani S., Vetrano R.M., Ceretti E., Ferraris E. (2022). Aerosol Jet® Printing of Poly (3, 4-Ethylenedioxythiophene): Poly (Styrenesulfonate) onto Micropatterned Substrates for Neural Cells In Vitro Stimulation. Int. J. Bioprint..

[21] Chietera F.P., Colella R., Verma A., Ferraris E., Corcione C.E., Moraila-Martinez C.L., Gerardo D., Acid Y.H., Rivadeneyra A., Catarinucci L. (2022). Laser-Induced Graphene, Fused Filament Fabrication, and Aerosol Jet Printing for Realizing Conductive Elements of UHF RFID Antennas. IEEE J. Radio Freq. Identif..

[22] Machiels J., Appeltans R., Bauer D.K., Segers E., Henckens Z., Van Rompaey W., Adons D., Peeters R., Geiβler M., Kuehnoel K. (2021). Screen Printed Antennas on Fiber-Based Substrates for Sustainable HF RFID Assisted E-Fulfilment Smart Packaging. Materials.

[23] Striani R., Stasi E., Giuri A., Seiti M., Ferraris E., Esposito Corcione C. (2021). Development of an Innovative and Green Method to Obtain Nanoparticles in Aqueous Solution from Carbon-Based Waste Ashes. Nanomaterials.

[24] Seiti M., Ginestra P.S., Verma A., Ceretti E., Ferraris E. (2022). Aerosol Jet® Printing on stereolithography resin substrates for in-vitro dual bioreactor sensing. Procedia CIRP.

[25] Borghetti M., Cantù E., Ponzoni A., Sardini E., Serpelloni M. (2022). Aerosol Jet Printed and Photonic Cured Paper-Based Ammonia Sensor for Food Smart Packaging. IEEE Trans. Instrum. Meas..

[26] Gibney R., Patterson J., Ferraris E. (2021). High-Resolution Bioprinting of Recombinant Human Collagen Type III. Polymers.

[27] Gibney R., Ferraris E. (2021). Bioprinting of collagen type I and II via aerosol jet printing for the replication of dense collagenous tissues. Front. Bioeng. Biotechnol..

[28] Ahmad J., Andersson H., Sidén J. (2019). Screen-Printed Piezoresistive Sensors for Monitoring Pressure Distribution in Wheelchair. IEEE Sens. J..

[29] Narakathu B.B., Eshkeiti A., Reddy A.S.G., Rebros M., Rebrosova E., Joyce M.K., Bazuin B.J., Atashbar M.Z. A novel fully printed and flexible capacitive pressure sensor. Proceedings of the Sensors, 2012 IEEE.

[30] Tiwana M.I., Redmond S.J., Lovell N.H. (2012). A review of tactile sensing technologies with applications in biomedical engineering. Sens. Actuators A Phys..

[31] Duan Y., He S., Wu J., Su B., Wang Y. (2022). Recent Progress in Flexible Pressure Sensor Arrays. Nanomaterials.

[32] Polzinger B., Keck J., Matic V., Eberhardt W., Kück H. (2020). D4.1-Inkjet and Aerosol Jet® Printed Sensors on 2D and 3D Substrates. AMA Conf..

[33] Rajala S., Schouten M., Krijnen G., Tuukkanen S. (2018). High Bending-Mode Sensitivity of Printed Piezoelectric Poly(vinylidenefluoride- co-trifluoroethylene) Sensors. ACS Omega.

[34] Ramalingame R., Hu Z., Gerlach C., Rajendran D., Zubkova T., Baumann R., Kanoun O. (2019). Flexible piezoresistive sensor matrix based on a carbon nanotube PDMS composite for dynamic pressure distribution measurement. J. Sens. Sens. Syst..

[35] Wang L., Ding T., Wang P. (2009). Thin flexible pressure sensor array based on carbon black/silicone rubber nanocomposite. IEEE Sens. J..

[36] Park S., Kim H., Vosgueritchian M., Cheon S., Kim H., Koo J.H., Kim T.R., Lee S., Schwartz G., Chang H. (2014). Stretchable Energy-Harvesting Tactile Electronic Skin Capable of Differentiating Multiple Mechanical Stimuli Modes. Adv. Mater..

[37] Zhu Y.J. (1992). Mechanical Modification of Elastomers–Filler Reinforcement and Blending.

[38] Kalantari M., Dargahi J., Kövecses J., Mardasi M.G., Nouri S. (2011). A new approach for modeling piezoresistive force sensors based on semiconductive polymer composites. IEEE/ASME Trans. Mechatron..

[39] Ruschau G.R., Yoshikawa S., Newnham R.E. (1992). Resistivities of conductive composites. J. Appl. Phys..

[40] Baranowski P., Janiszewski J., Małachowski J. (2017). Tire rubber testing procedure over a wide range of strain rates. J. Theor. Appl. Mech..

[41] Niemczura J.G. (2009). On the Response of Rubbers at High Strain Rates.

[42] Tang Z., Jia S., Zhou C., Li B. (2020). 3D Printing of Highly Sensitive and Large-Measurement-Range Flexible Pressure Sensors with a Positive Piezoresistive Effect. ACS Appl. Mater. Interfaces.

